# Supplementary material for: Cell-guiding microporous hydrogels by photopolymerization-induced phase separation
Source: Nat Commun. 2025 May 27;16:4923. doi: 10.1038/s41467-025-60113-9 (PMC12116776; doi:10.1038/s41467-025-60113-9)
Supplement: Supplementary file 1 — Supplementary Information [file 41467_2025_60113_MOESM1_ESM.pdf]

Supplementary Information

Cell-guiding Microporous Hydrogels by Photopolymerization-induced Phase Separation

Monica Z. Müller[1], Margherita Bernero*[1], Chang Xie[1], Wanwan Qiu[1,2], Esteban Oggianu[1], Lucie Rabut[1], Thomas C.T. Michaels[2,3], Robert W. Style[4], Ralph Müller[1] & Xiao-Hua Qin[1,2]*

[1]Institute for Biomechanics, ETH Zurich, Zurich, Switzerland.

[2]Bringing Materials to Life Initiative, ETH Zurich, Switzerland.

[3]Institute of Biochemistry, ETH Zurich, Zurich, Switzerland.

[4]Laboratory for Soft Materials and Interfaces, ETH Zurich, Zurich, Switzerland.

*denotes equal contribution

Correspondence should be addressed to X.Q. (email: qinx@ethz.ch)

Content:

- Supplementary Methods
- Supplementary Figures 1-20 and Tables 1-4
- MATLAB Script for FFT Analysis
- Supplementary Movies 1-4

Supplementary Methods

Hydrogel Precursor Preparation

In some experiments, a "10%" DS stock solution diluted by volume from a 50% stock solution was used. However, the density of the 50% stock solution was shown to be around 1.25 g/mL (Supplementary Fig. 18), so that the concentration of the diluted "10%" stock solution and so also the final DS concentrations in the hydrogel precursor mixture were higher than anticipated. The slightly higher average density of the diluted 10% stock solution compared to a directly prepared 10% stock solution also hints at this. In this report, 2.5% DS and 3% DS with 2% nPVA refer to compositions prepared from the diluted 10% DS stock solution, which correspond to actual concentrations of around 3.125% and 3.75% DS respectively as calculated by the density measurements.

Synthesis of nPVA

Norbornene-functionalized polyvinyl alcohol (nPVA) was synthesized as described previously[1]. First, 8.01 g of PVA (Sigma-Aldrich 10853, M_W 47 kDa; 182 mmol -OH groups, 1 equiv.) were dissolved in 130 mL anhydrous DMSO (Sigma-Aldrich 276855, ≥99.9% purity) at 60 °C under stirring and argon protection. The mixture was distilled under vacuum to remove ~10% of solvent. A solution of p-Toluenesulfonic acid (Thermo Scientific, 99% purity; 29 mg, 0.3 wt% of PVA) in anhydrous DMSO was added under argon protection. Then, cis-5-norbornene-endo-2,3-dicarboxylic anhydride (abcr AB147239, 97% purity; 2989 mg, 18.2 mmol, 0.1 equiv.) dissolved in anhydrous DMSO was added to the mixture dropwise. The reaction was stirred for 24 h at 55 °C. Finally, the product was dialyzed (M_W cutoff: 6-8 kDa) against distilled water for 24 h, aqueous $NaHCO_3$ at pH 8 for 12 h and finally MilliQ water for another 24 h. After lyophilization, a white solid was obtained and analyzed by 400 MHz ^{1}H-NMR in D_2O (degree of functionalization: 3.5%) as shown in Supplementary Fig. 19.

Synthesis of nPVA-RGD Conjugates

Cell-adhesive RGD-modified nPVA conjugates were synthesized by radical-mediated thiol-ene photoclick conjugation as previously described[1]. Briefly, 200 mg of nPVA (degree of substitution, 7%) was dissolved in 2 mL of reaction buffer to reach a final concentration of 10%. The reaction buffer contains PBS (1x, pH 7.40) and 0.05% LAP photoinitiator. To this solution, 20.6 mg of CGRGDSP peptide (China Peptides, 98.15% purity; 0.12 eq.) dissolved in PBS (200 µL) was added and mixed using a magnetic stirrer. Then, UV-365 nm irradiation was applied to initiate photoconjugation at a light intensity of 40 mW/cm^2 for 90 min. Finally, the mixture was purified by dialysis (M_w cut-off: 6-8 kDa) in deionized water for 48 hours.

After lyophilization, the nPVA-RGD conjugates were obtained as white solids (yield = 85%). ^{1}H-NMR spectrum (D_2O) of nPVA-RGD conjugates is shown in Supplementary Fig. 20.

nPVA-DS PIPS Composition

The compositions used are described in detail in Supplementary Tables 2-4. The components were added in the order listed, where it was especially important that the crosslinker is added last. For cell embedding, the order of mixing was altered to minimize the time that cells are outside of their medium (Supplementary Table 4). For mixes 1-3, stock solutions were prepared using weight concentrations (w/w) to correlate with the approximated phase diagram. For practicability of further experiments with mixes 4-6, stock concentrations were changed to weight per volume (w/v).

The (Bio)resin Based on nPVA-DS-Gelatin for Volumetric Printing

Gelatin was added because it gelates at lower temperatures, which makes it suitable to prevent sedimentation in volumetric printing. Gelatin was dissolved at 40 °C for 2-3 hours for a stock solution of 10% (w/v). The composition used for acellular printing experiments is described in Supplementary Table 3. The components were mixed in this order and were warmed to prevent gelation during mixing. For volumetric bioprinting, the cells were resuspended in the gelatin fraction and added to the mix last.

Gel Casting

PDMS molds were prepared from 0.5-1 mm thick sheets by punching out wells using biopsy punches. Well diameters of 3 mm were used for acellular experiments while cells were embedded in wells of 4 mm (imaging) or 8 mm (ALP assay). For cell experiments, the molds were sterilized by soaking in 70% ethanol overnight. The molds were attached to a coverslip (30 mm Ø) or into a No. 1.5 glass bottom confocal dish (VWR). The wells were filled with precursor solution and Sigmacoated coverslips were placed on top of the mold. After curing, PBS was added to the edge of the glass to facilitate loosening of the slide. The hydrogels were then transferred with a plastic spatula to a 12-well or 24-well plate filled with 1 mL of PBS for swelling.

The gels were cured for 5 min under UV lamps with an intensity of 10-20 mW cm^{-2}. The LAMAG UV lamp (365 nm) was used for most acellular experiments and the LEDD1B Thorlabs UV lamp (365 nm) for tuning the light intensity and cell embedding experiments. After curing, the sample was directly imaged. Washed samples, however, were imaged after at least 2 days incubation in PBS at 4 °C. For the intensity tuning experiment, the gels with the light intensities 5 and 10 mW cm^{-2} were cured for 20 and 10 min respectively to ensure complete

crosslinking. A power meter (S120VC, Thorlabs) with a sensor (PM100D S12VC, Thorlabs) was used to measure the light intensities.

PIPS Composition Screening

The phase states of various hydrogel compositions were characterized by preparing the hydrogels in a µ-Plate 384-well Glass Bottom (ibidi). Hydrogels were made by sequentially mixing stock solutions of nPVA, LAP, DS, and PEG-2-SH. The proportions of each component were adjusted to achieve the target composition, with the final total volume of the mixture set to 40 µL. A 12 µL aliquot of the final mixture was added to each triplicate well of the µ-Plate. Then, the samples were imaged using an inverted transmitted-light Primovert (Zeiss) microscope equipped with an LD Plan-Achromat 20x/0.3 Ph1 objective and IDS Peak Cockpit 2.1.0.0 software (IDS Imaging Development Systems GmbH). The mixture was subsequently UV-cured for 5 minutes using a Thorlabs UV lamp with a light intensity of 10 mW/cm². After curing, the samples were imaged again.

For compositions within the 2-3.5% nPVA and 2-3.5% DS range, stock solution concentrations were as reported in Supplementary Table 4. However, to allow a broader screening of compositions, the PEG-2-SH stock solution concentration was increased to 30% (w/v) for phase state characterization of more extreme compositions. To maintain consistency with the 16.51% PEG-2-SH stock solution, phase states of samples in the narrower composition window were also evaluated using the 30% PEG-2-SH stock solution. Most compositions showed similar phase states regardless of the PEG-2-SH stock concentration used. However, the 3/3% nPVA/DS composition, which is close to the binodal curve, exhibited sensitivity to environmental factors such as stock solution concentration. This resulted in different phase states, with phase-separating (PIPS) behavior observed when using the 16.51% PEG-2-SH stock solution, and a homogeneous phase state (PS) observed when using the 30% PEG-2-SH stock solution.

Swelling Ratio

Hydrogel discs (diameter, 6 mm; height, 2 mm) were swollen in PBS at 37 °C for two days, the equilibrium wet mass (M_w) and the dried mass (M_d) of each gel disc were recorded, respectively. A sample size of 3-4 was used. The mass swelling ratio (Q_m) was calculated as:

$$Q_m = M_w/M_d$$

Photo-Rheology

In situ photo-rheological measurements were performed on a modular photo-rheometer (Anton Paar MCR 302) equipped with a 20 mm parallel plate geometry, a glass floor and UV-LED

lamp (Thorlabs, Germany). The tests were carried out at room temperature (22 °C) unless otherwise noted. Mineral oil was loaded surrounding the sample to prevent it from drying during testing. The time-sweep of shear storage and loss modulus were collected under irradiation of 365 nm UV-LED lamp (20 mW cm^{-2}) in triplicates at 0.5 % strain and 1 Hz frequency with 100 µm gap. Frequency and amplitude sweeps were performed to assess the mechanical behavior of the hydrogels after curing. Right after the UV crosslinking measurement, the frequency sweep was done *in situ* with an angular frequency of 0.1-100 rad/s and a strain of 0.5%. Amplitude sweeps were performed with an angular frequency of 10 rad/s and a strain of 0.01-100% or up to 1'000%. For the previously cured and swollen hydrogels, a parallel plate with 10 mm diameter was used and the gap was set to have a normal force of around 0.1 N.

3D Pore Analysis

To quantify the 3D pore characteristics, rhodamine-labelled hydrogels were imaged by confocal microscopy using the Zeiss LSM 780 with a 63× objective. The 40 µm z-stacks were processed and analyzed following the protocol by Vandaele et al.[2] using their MATLAB script from a public GitHub repository (https://github.com/BorisLouis/Segmentation). The images within a stack were segmented with an adaptive threshold sensitivity of 0.6, then pores were identified using a watershed algorithm. The porosity was directly obtained while 3D external pore radii and pore connectivity (number of adjacent pores) were computed by fitting spheres into the pores in 3D. We excluded pores with a radius below 0.75 µm (diameter of 1.5 µm) from our further analysis due to the limits of resolution from our image acquisition method.

For the 3D analysis of pores in the cellular hydrogels, 25 µm confocal z-stacks of the FITC-dextran (500 kDa) perfused pore space were acquired with a 40× objective, and the image signal was inverted in the analysis script.

hMSC Cell Expansion and Passaging

Human bone marrow-derived mesenchymal stromal cells (hMSCs) were purchased from Lonza (PT-2501, Lot# 19TL281098, 25 year-old male). Frozen cells were thawed rapidly at 37°C and suspended in Dulbecco's Modified Eagle's medium (DMEM) at 4°C. While centrifuging the cells at 300g for 10 min at 4°C, 90 mL expansion medium (DMEM with 10% fetal bovine serum (FBS), 1% antibiotic-antimycotic (Anti-Anti), 1% non-essential amino acids and 0.001% bFGF aliquot) was loaded per triple flask. 10 mL of the cell suspension was then added to the flask. Incubation was done at 37°C with 5% CO_2. On day 3 (d3), 50 mL expansion medium was added and on d5, the medium was changed with 100 mL per flask. The cells were split when they reached 80% confluence. First, the cells were washed twice with PBS at 37°C. Then,

15 mL of 0.05% trypsin-EDTA was loaded. After incubation at 37°C for 4 min and tapping the flask to detach all cells, 15 mL control medium (DMEM with 10% FBS and 1% Anti-Anti) was added per flask and the cell suspension transferred to a falcon tube. After an additional washing step with 15 mL control medium, the cells were again centrifuged for 10 min at 4°C. The cells were then resuspended in 10 mL control medium at 4°C and counted using a hemocytometer and trypan blue. Finally, the cells were resuspended at the desired concentration after another centrifugation step in expansion medium and loaded into a fresh cell culture flask. Medium was changed three times a week thereafter and the cells again split once confluence was reached.

HDF Cell Expansion and Passaging

Human dermal fibroblasts (HDFs) were purchased from Cell Applications Inc. (106-05a, Lot# 1526, 57-year-old female) and expanded under standard cell culture conditions. Cells were maintained in tissue culture-treated flasks using DMEM supplemented with 10% FBS and 1% Anti-Anti at 37°C in a humidified atmosphere containing 5% CO_2. Culture media were replaced every 2–3 days to ensure optimal cell growth. Upon reaching 70–90% confluency, HDFs were passaged using a standardized protocol. Briefly, cells were washed with sterile PBS to remove residual serum and detached by incubating with 0.05% trypsin-EDTA at 37°C for 2–5 minutes. Trypsinization was halted by adding an equal volume of complete medium. The cell suspension was centrifuged at 200 g for 5 minutes, and the pellet was resuspended in fresh medium. Cells were then seeded into new culture vessels at a predetermined density for further expansion. All procedures were conducted under aseptic conditions to maintain culture integrity.

Live/Dead Assay

After washing with warm PBS, a staining solution containing Calcein AM (1:500) and ethidium homodimer-1 (EthD-1, 1:1000) was loaded. The sample was then incubated for 15 min and washed twice with PBS. With the sample in PBS, the dish was sealed with parafilm and imaged within one hour. Automated particle analysis was performed in ImageJ on maximum intensity projections (MIPs) of the live and dead channels. Viability was calculated as the number of live cells divided by the sum of all cells.

Fixation and Actin-Nuclei Staining

The samples were fixed with 4% PFA by washing with warm PBS, incubating with 4% PFA for 15 min at RT and washing twice with PBS. PBS was then added for hydration and the sample stored at 4 °C until the staining procedure was started. For the staining, the sample was first blocked with 1% BSA for 1 h and then permeabilized in 0.2% Triton X-100 for 10 min. After washing thrice with PBS, a staining solution was added, which contained 0.1% BSA,

Phalloidin 488, 555 or 647 and Hoechst (1 mg/mL) at dilutions of 1:400 and 1:1000 respectively. After 1-2 hours of incubation at RT protected from light, the sample was washed thrice with PBS and PBS was loaded for hydration.

Cell Area Quantification

For the cell area quantification, MIPs were segmented and analyzed. The threshold was manually set and kept the same for all groups within one experiment or determined using the mean of the grey levels (Mean thresholding method). The area was measured by the actin signal. The cell number was determined by counting the nuclei particles. Unless otherwise noted, the average cell area was then calculated as the total area divided by the cell number for each z-stack.

Osteocalcin Immunostaining

The hMSC samples were fixed as described above and cryosectioned. The hydrogels were first cryoprotected in 30% sucrose in PBS at 4°C overnight, then in a 1:1 mixture of 30% sucrose and Tissue-Tek O.C.T. Compound for 3 h at 4°C. The triplicates were transferred into a cryomold, covered in O.C.T. Compound and frozen in liquid nitrogen. Finally, the samples were cryosectioned on a CryoStar NX70 Cryostat with block temperature at -12°C, blade temperature at -20°C and a section thickness of 20 µm. On the day of staining, the sections were thawed, washed by dipping in milliQ water and permeabilized in 0.2% Triton X-100 for 10 min. After washing thrice with PBS, samples were blocked for 1 h with 5% donkey serum (secondary antibody host) in 1% BSA before being incubated with the anti-Ocn primary antibody (rabbit polyclonal, Abcam, ab93876, diluted 1:200) in 1% BSA at 4°C overnight. The secondary antibody (donkey anti-rabbit IgG H&L Alexa Fluor 647, Abcam, ab150075, diluted 1:500) was incubated together with Phalloidin 555 (1:400) and Hoechst (1:1000) in 1% BSA for 2h at RT, protected from light. After washing thrice with PBS, the sections were covered with a drop of Prolong Gold Antifade mounting medium and a glass coverslip, and confocal z-stacks were acquired at the Zeiss LSM 780.

Collagen I Immunostaining

For bulk staining of 3D HDF cultures, samples were fixed and permeabilized in triplicates as described above for actin-nuclei staining. To detect Col 1, the gels were incubated with an anti-Collagen I primary antibody (mouse monoclonal, Abcam, ab6308, diluted 1:200) at a concentration of 3.5-7 µg/ml in 1% BSA for 1 hour at room temperature. Afterwards, the gels were washed three times with PBS to remove unbound primary antibodies and then incubated with a fluorescently labeled secondary antibody (donkey anti-mouse IgG H&L Alexa Fluor 488, Invitrogen, A21202, diluted 1:500) for 30 minutes in the dark. Samples were then

counterstained with Hoechst only or Hoechst and phalloidin 647 as described above. Following another set of PBS washes, confocal fluorescence microscopy (Zeiss LSM 780) was used to visualize the Col 1 distribution within the gels.

Alkaline Phosphatase (ALP) Assay

To quantify ALP activity as another marker for osteogenic differentiation in hMSCs, a colorimetric ALP Assay Kit (Abcam, ab83369) was used. Cell-laden hydrogel samples (8 mm diameter, 220’000 cells in theory) were collected in triplicates at each time point. In addition to the 3D samples, 3× 200’000 cells were collected on day 0 before cell embedding as a baseline control. All samples were washed with PBS and resuspended in 300 µL ALP assay buffer before being homogenized using autoclaved pellet pestles and the pestle motor, flash frozen in liquid nitrogen and stored at -80 °C.

The ALP assay was performed following the manufacturer’s instructions and as described previously[3]. In short, samples were thawed on ice and centrifuged to obtain the supernatant without any insoluble materials. The assay was performed for each sample in technical triplicates with a background control, and standard dilutions in duplicates. 50 µL of the sample supernatant as well as the corresponding amounts of 5 mM *p*-nitrophenyl phosphate (pNPP) substrate solution and ALP assay buffer were added to each sample well. The standard curve and background wells were prepared as instructed. After 1h of incubation at RT in the dark, the stop solution was added and absorption at 405 nm was measured on a Tecan Spark 10M plate reader. The sample measurements were first corrected by the blank and the background control and finally, ALP activity was quantified based on the standard curve.

OsteoImage Staining for Mineralization

Bulk hydrogel samples were fixed as described above and stained using the OsteoImage Mineralization Assay (Lonza, PA-1503) to specifically stain hydroxyapatite mineral. A solution of OsteoImage reagent (1:100) and Hoechst 33342 (1:200) was prepared in 0.1% TritonX-100 in PBS and incubated with the samples for 30 min in the dark. Afterwards, the hydrogels were washed 3× 5 min with PBS and hydrated with PBS for imaging on the Zeiss LSM 780 confocal microscope. For OsteoImage quantification, MIPs were segmented with a manually set threshold and the percentage of OsteoImage-positive area was determined via particle analysis.

Volumetric (Bio)-Printing

VP was performed on a Tomolite 1.0 bioprinter (Readily3D) together with the software Apparite. First, a laser dose test was conducted, in which defined spots were irradiated with varying laser intensity and irradiation time. After irradiation, it was noted which spots were polymerized. A second test with a smaller range was then conducted to determine the threshold

needed for polymerization. For volumetric printing, 3-4 mL of precursor solution was loaded into 18 mm diameter Pyrex vials. A refractive index of 1.37 was chosen and the peak-to-average ratio was set to 6:1. The optimal settings as calculated by the software were used.

Imaging and Image Analysis

The confocal microscopes Leica SP8 MP, Leica SP8 AOBS CARS and Zeiss LSM 880 Airyscan were used for imaging. For imaging the whole printed constructs, a stereomicroscope (Leica Stereo) was used. Microscopes were used with the software LAS X (Leica) or ZEN 2012 (Zeiss). Images and videos during the volumetric printing process were captured with the integrated camera of the volumetric printer. Image analysis was performed in Fiji.

Supplementary Figure 1

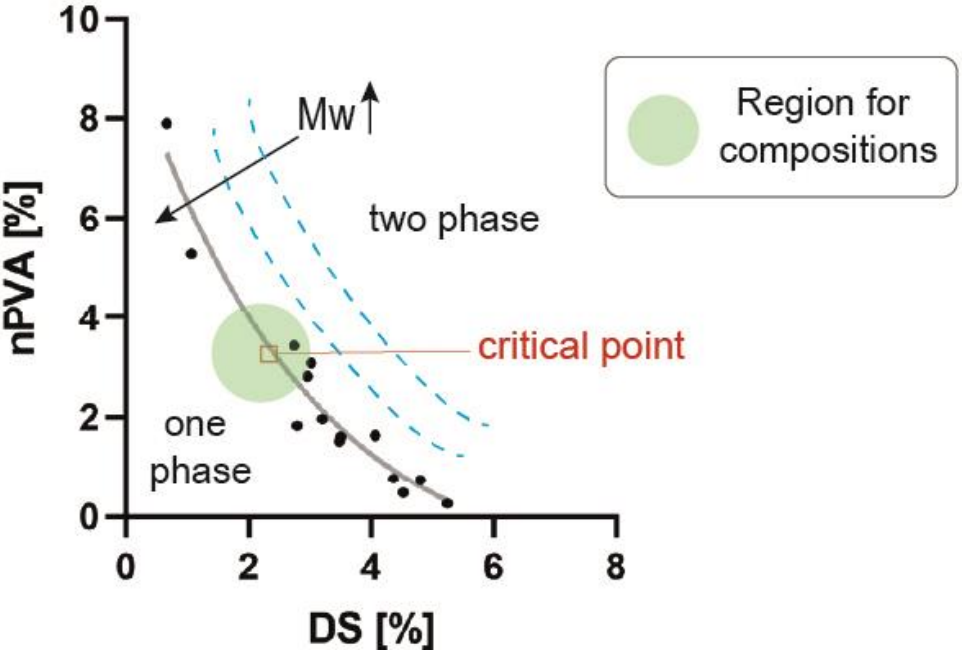

Supplementary Fig. 1: Schematic showing the general rationale for precursor composition selection and the expected binodal shift during PIPS. It presents a plot of two polymer concentrations A (nPVA) and B (DS) plotted against each other accompanied by the corresponding binodal curve. This curve demarcates the boundary between the one-phase region and the two-phase region. As molecular weight increases during photocrosslinking, the binodal shifts towards lower concentrations, approximately crossing the region in which hydrogel precursor compositions are chosen, resulting in an observable phase transition. Just above the critical point the volume of both phases is theoretically equal.

Supplementary Figure 2

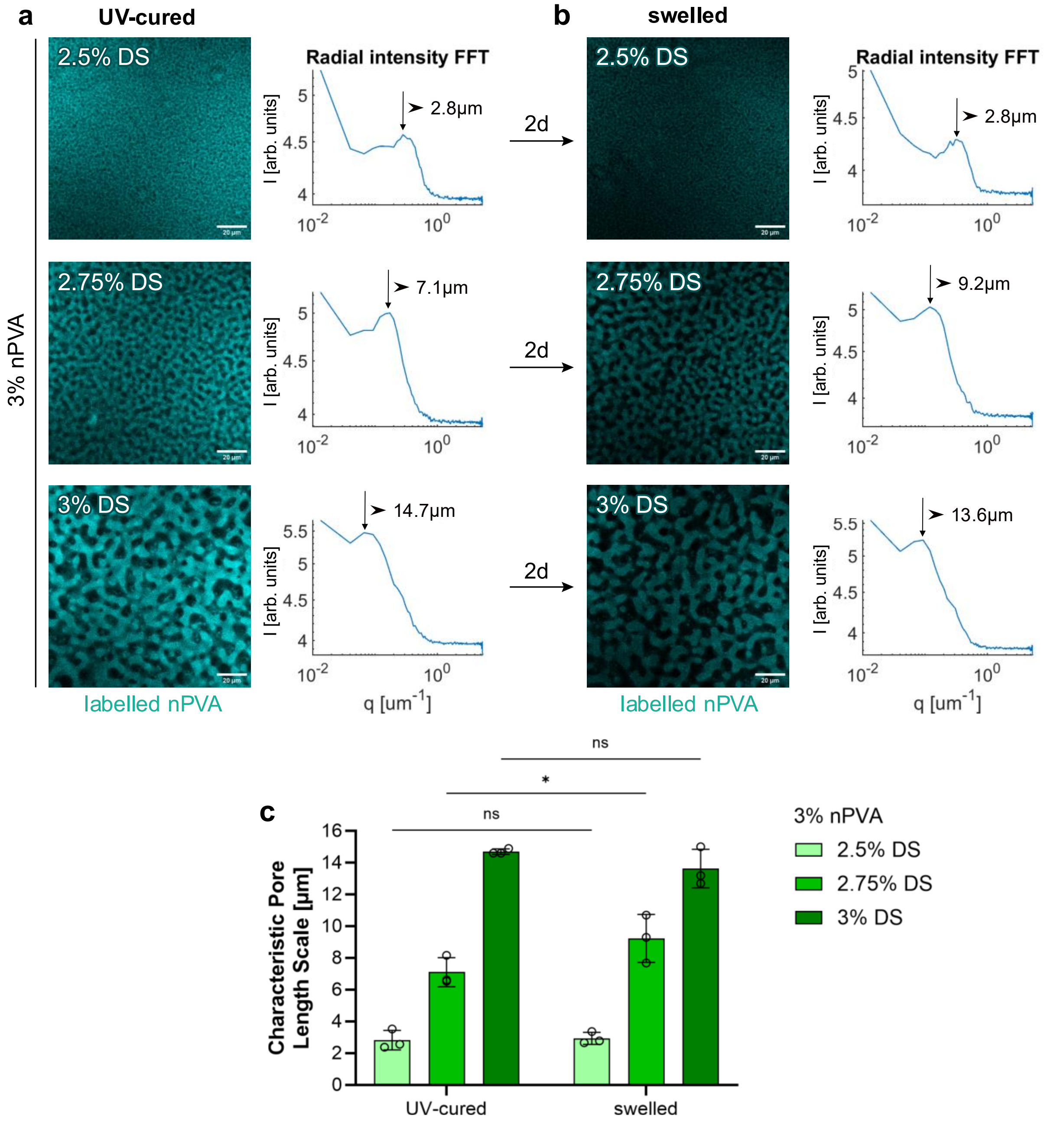

Supplementary Fig. 2: RIFFT plots of microporous hydrogels. a Hydrogels after UV crosslinking show a peak (arrow) corresponding to different length scales before swelling as indicated. **b** The same hydrogels still show similar peaks and pore length scales after swelling for 2 days. **c** No significant differences were found before and after swelling, except for the increasing length scale in 3% nPVA + 2.75% DS (N=3 samples, two-way ANOVA/Šídák between UV-cured and swelled). *: p=0.0429. Data presented as mean ± SD. Scale bars: 20 μm.

Supplementary Figure 3

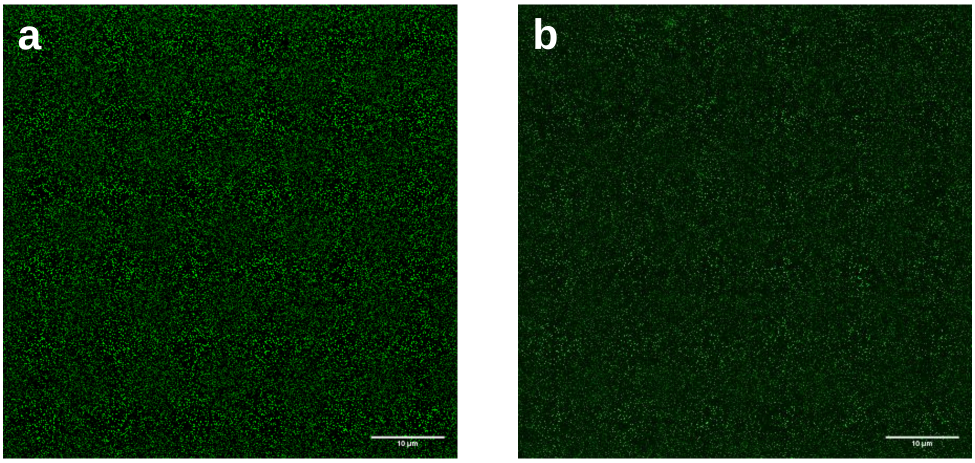

Supplementary Fig. 3: Hydrogels with lower DS concentrations showing no pores. 2% (w/w) nPVA (54 kDa, FITC-labelled nPVA : unlabelled nPVA = 1:50), with PEG-2-SH as crosslinker (thiol/ene ratio = 1.6); crosslinked with UV lamp (LAMAG); 2% (**a**) and 0% (**b**) (w/w) DS; unwashed; scale bars: 10 µm.

Supplementary Figure 4

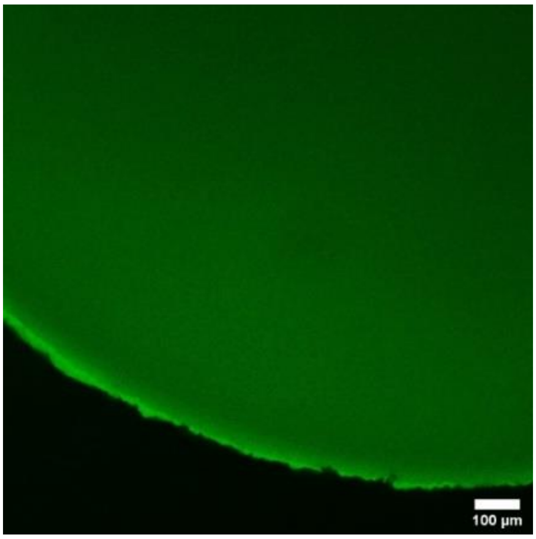

Supplementary Fig. 4: Control gel without DS showing no pores. CLSM images of 2% nPVA gels without DS after 3 days of equilibration in PBS and 2 days in FITC-dextran showing no pores.

Supplementary Figure 5

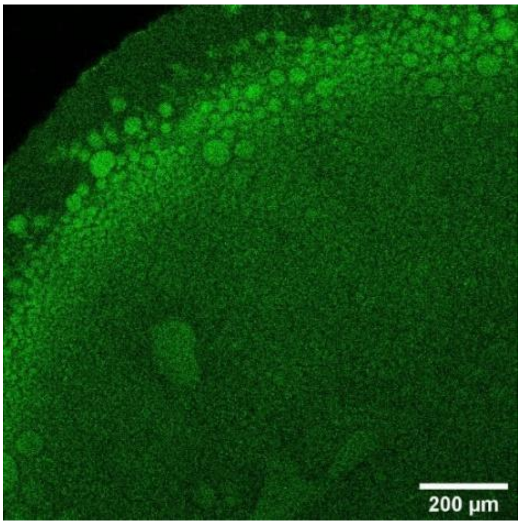

Supplementary Fig. 5: Inhomogenously sized blobs indicating phase separation before photopolymerization with 3% nPVA 3.5% DS. After UV curing (18 mW cm^{-2}, 365 nm), a 1/50th of FITC-labelled nPVA (green) was used for confocal imaging.

Supplementary Figure 6

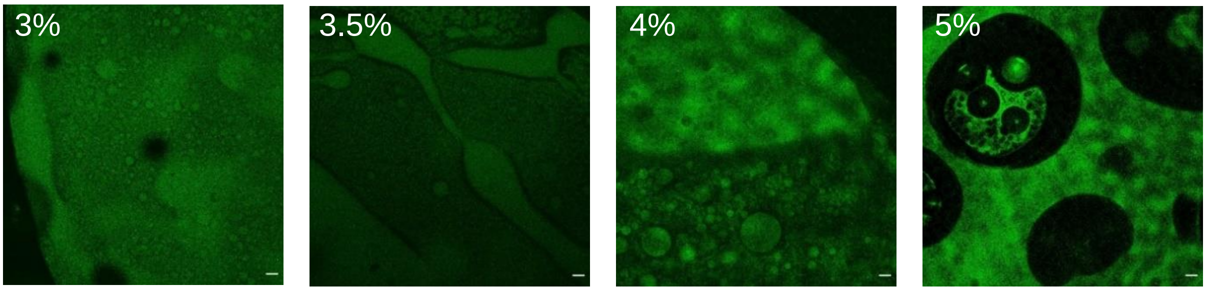

Supplementary Fig. 6: Hydrogels with higher nPVA content showing inhomogeneous morphologies. CLSM images of hydrogels with different concentrations of nPVA (54 kDa, 1:50 FITC-labelled nPVA : unlabelled nPVA), with PEG-2-SH as crosslinker (thiol/ene ratio = 1.6); crosslinked with UV lamp; 2.5% (w/w) DS; unwashed; 3%, 3.5%, 4%, 5% (w/w) nPVA. Scale bars: 50 µm.

Supplementary Figure 7

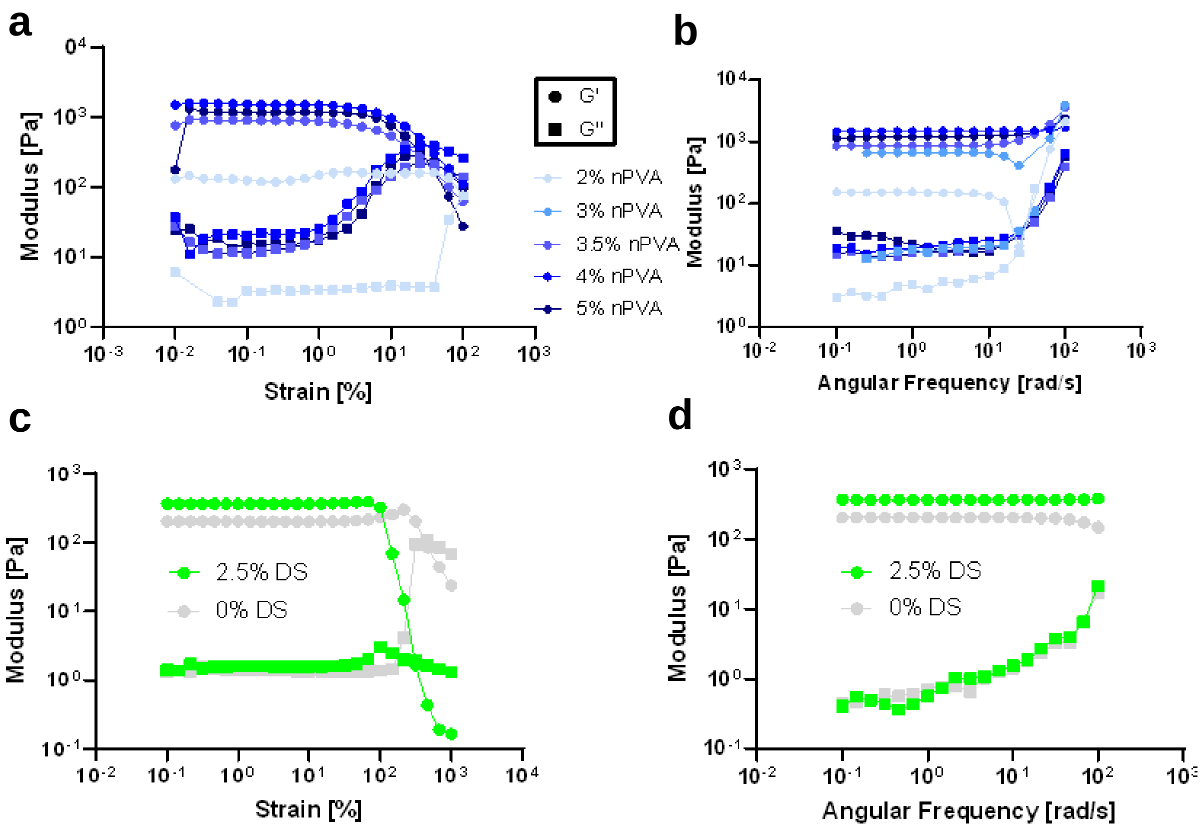

Supplementary Fig. 7: Amplitude and frequency sweeps of various nPVA-DS hydrogels after photo-rheometry. a,b The amplitude (**a**) and frequency (**b**) plots of hydrogel samples formed with varying nPVA% and 2% DS. **c, d** The amplitude (**c**) and frequency (**d**) plots of hydrogel samples formed with 0 vs. 2.5% DS and 2% nPVA. PEG-2-SH was used as a crosslinker (thiol/ene = 1.6). Amplitude sweeps: 10 rad/s angular frequency and 0.01 - 100% strain; frequency sweeps: 0.5% strain and 0.1-100 rad/s angular frequency.

Supplementary Figure 8

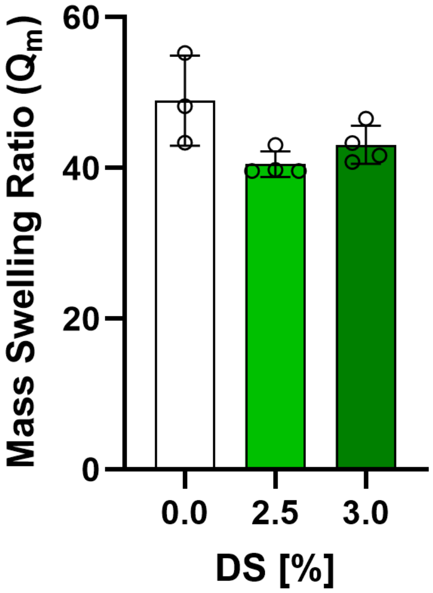

Supplementary Fig. 8: Equilibrium mass swelling ratio of hydrogels formed with 2% nPVA and varying DS content after two days of swelling in PBS. N = 3 samples for 0% DS and N = 4 samples for 2.5%/3% DS. Data presented as mean ± SD.

Supplementary Figure 9

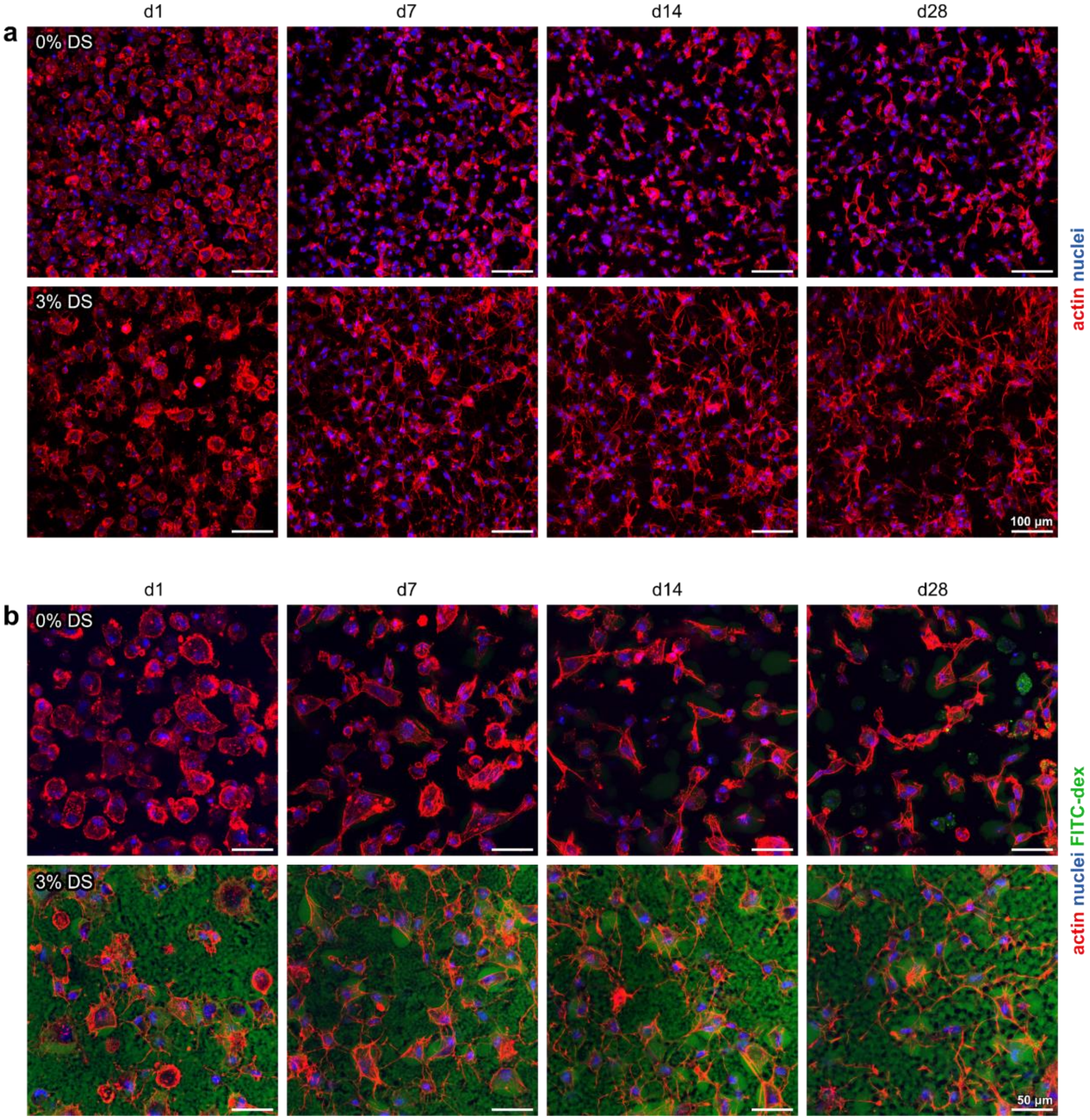

Supplementary Fig. 9: Morphology of hMSCs in osteogenic 3D culture. a Representative MIPs of embedded hMSCs stained with Hoechst (nuclei) and phalloidin (actin) on day 1, 7, 14 and 28. **b** Higher magnification MIPs of actin-nuceli-stained cells overlaid with the FITC-dextran-perfused pore space, evidencing the high porosity of 3% DS hydrogels compared to 0% DS control samples.

Supplementary Figure 10

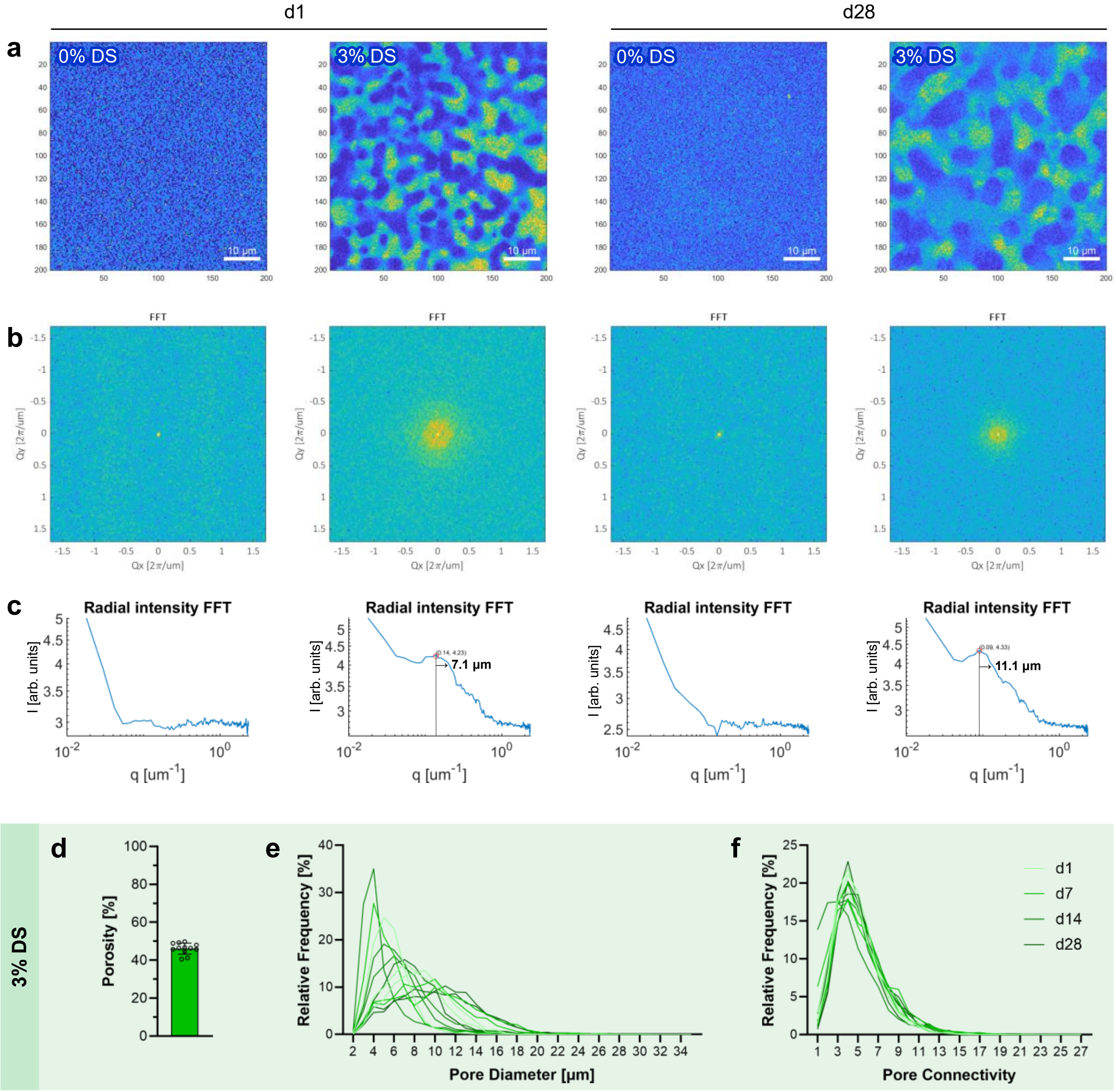

Supplementary Fig. 10: Pore quantification of cellular hydrogels. a-c Pore length scale quantification pipeline using RIFFT. **a** Representative confocal images of fixed, cellular gels perfused with FITC-dextran (500kDa). ROIs were selected to exclude cells, as they are situated within larger, irregular cavities (see also Supplementary Fig. 9). **b** 2D Fast Fourier Transforms (FFT) of the corresponding images shown in the same column above. **c** Radial intensity plots of the FFT above show a peak if a particular signal frequency is especially prominent in the image. The characteristic pore length scale in the 3% DS conditions is marked alongside the peak. **d-f** Results of the 3D pore analysis in cellular hydrogels fixed throughout the culture period: **d**) porosity, **e**) pore size distribution determined from the external radii, and **f**) pore connectivity, defined as number of directly adjacent pores. Pore sizes below 1.5 µm were excluded due to the resolution limit. Bin size: 1.

Supplementary Figure 11

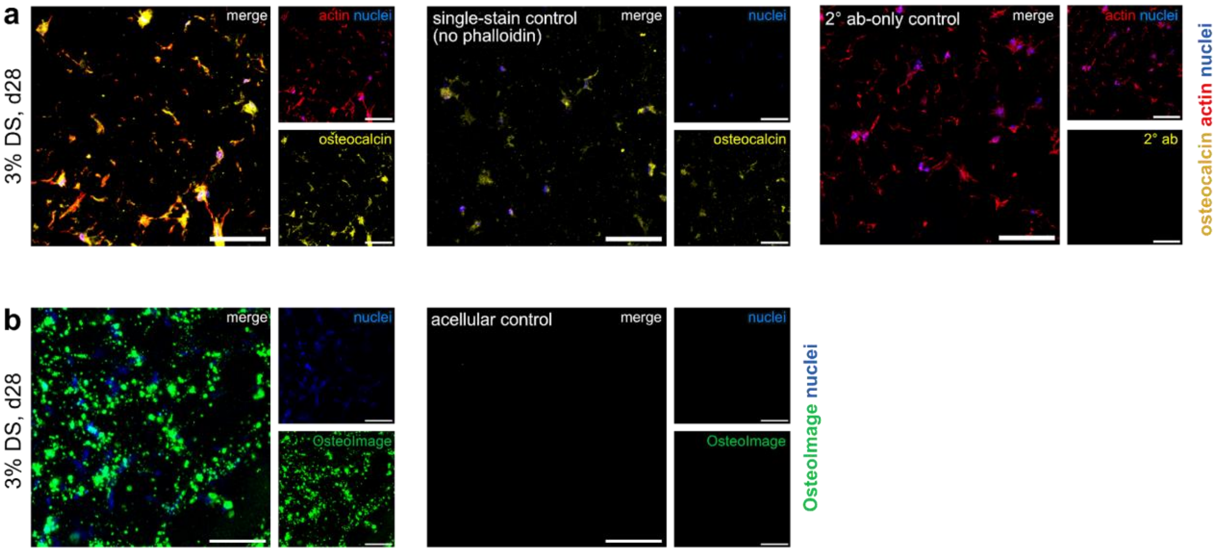

Supplementary Fig. 11. Staining controls for osteogenic markers of hMSCs encapsulated in the PIPS hydrogels. a Combined osteocalcin-actin-nuclei staining is compared to a single-stain control without phalloidin for bleed through of the actin signal into the osteocalcin channel and a 2° ab-only control for unspecific binding of the 2° ab. **b** The OsteoImage-nuclei staining for mineralization in cellular samples is compared to an acellular control sample on d28.

Supplementary Figure 12

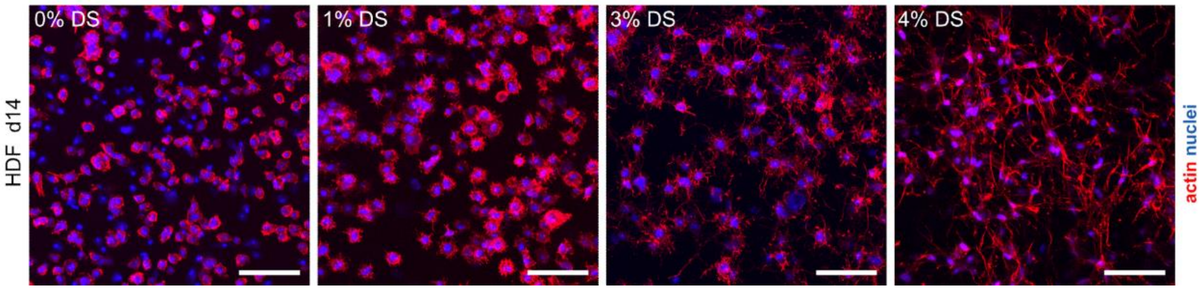

Supplementary Fig. 12. Confocal images of actin-nuclei-stained HDFs after 14 days of culture as an influence of DS concentration. Scale bars: 100 µm.

Supplementary Figure 13

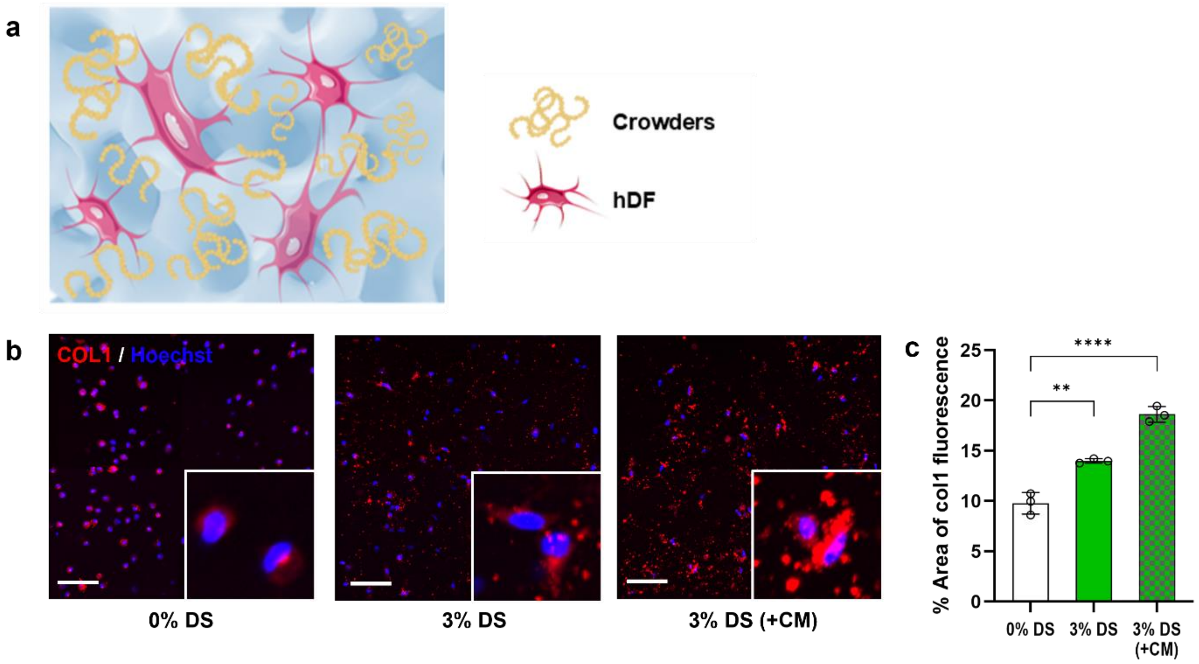

Supplementary Fig. 13. **a** Schematic of macromolecular crowding culture in PIPS hydrogels. **b** Confocal images of Collagen1-nuclei-stained HDFs (single-stain control) after 1 week of culture as an influence of DS concentration and crowder. **c** Quantification of the immunofluorescence of collagen 1 (N=3 samples, one-way ANOVA/Tukey). **: p=0.001; ****: p<0.0001. Data shown as mean ± SD. Scale bars: 100 µm.

Supplementary Figure 14

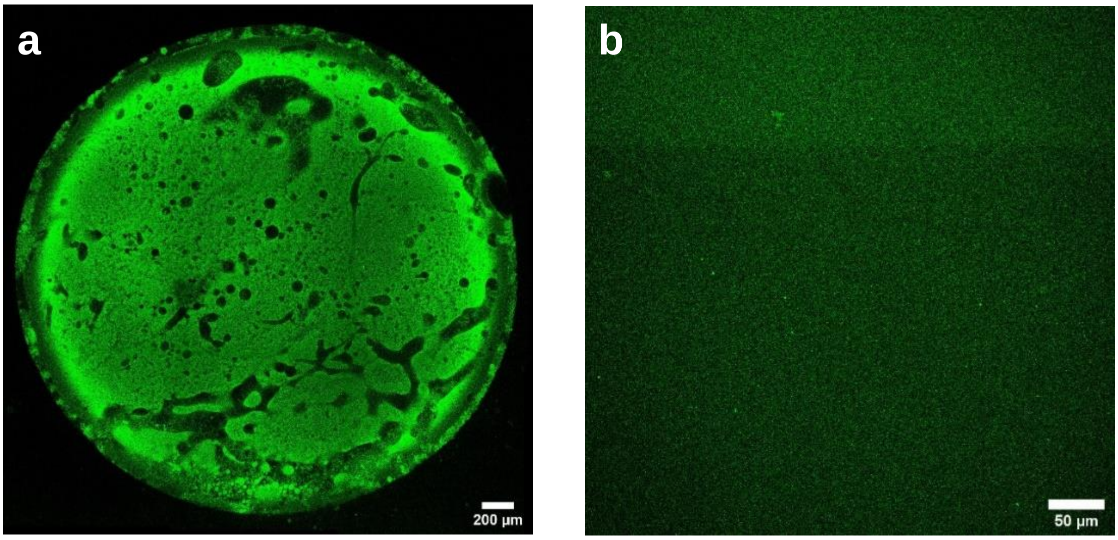

Supplementary Fig. 14: 1.5% DS composition resulting in inhomogeneous pores and 0.5% DS composition resulting in no pores. Confocal images of 2% nPVA 5% gelatin gels after washing with 1.5% DS showing large scale phase separation (**a**), 0.5% DS showing no phase separation at this resolution (**b**); a 1/50th of FITC-labelled nPVA (green) was used for confocal imaging.

Supplementary Figure 15

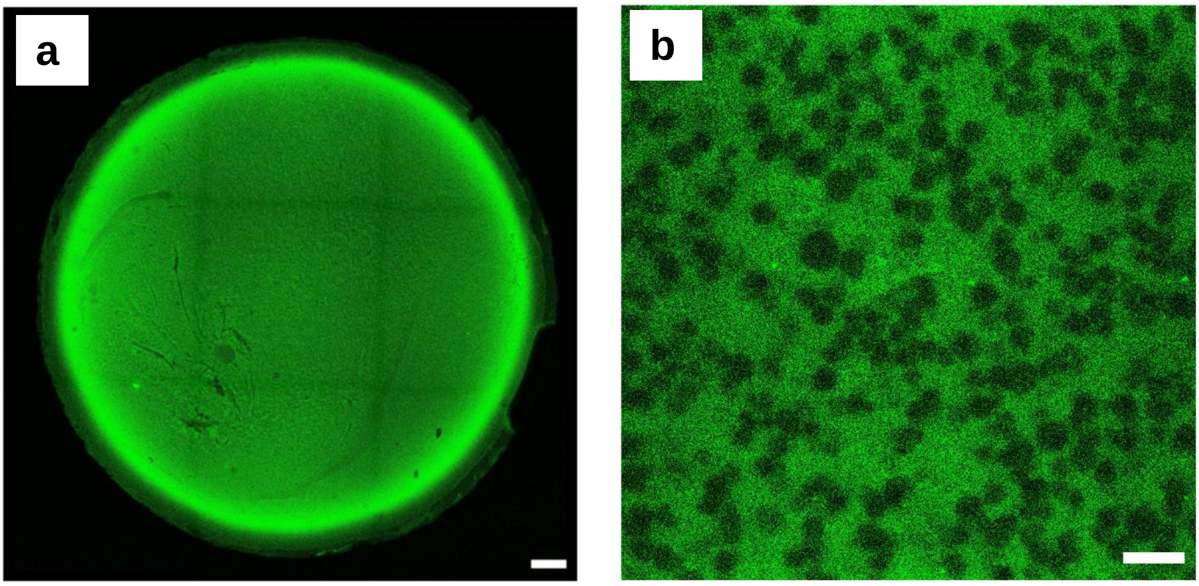

Supplementary Fig. 15: Pore imaging using the optimized composition with gelatin. Representative CLSM images of 2% nPVA 5% gelatin 1% DS gel after UV curing (18 mW cm^{-2}, 365 nm) in cold state and incubating in PBS at 37°C for over one hour to release gelatin. A 1/50th of FITC-labelled nPVA (green) was used for confocal imaging. **a** Whole well with almost complete homogeneity (merged image). **b** Gel at higher magnification within a homogenous region showing micropores. Scale bars: 200 µm (**a**) and 10 µm (**b**).

Supplementary Figure 16

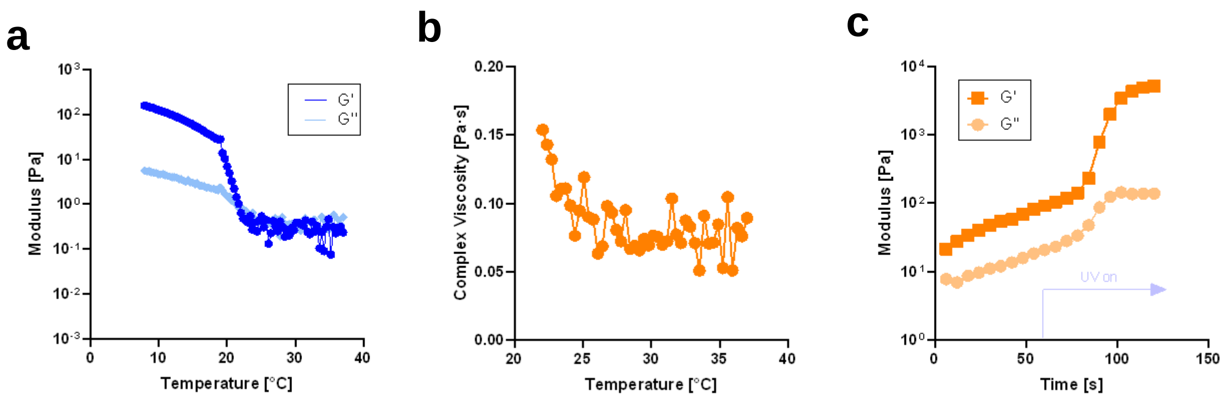

Supplementary Fig. 16: Temperature-dependent gelation, complex viscosity and crosslinking of the resin. **a** Temperature sweep of storage (G’) and loss (G’’) moduli from 37 °C to 8 °C showing the temperature-dependent gelation of gelatin at around 20-22 °C. **b** Complex viscosity obtained from the same measurements. **c** Time sweep of G’ and G’’ at 25 °C showing crosslinking after UV light (7.9 mW cm^{-2}, 365 nm) is turned on after 60 s of measuring. 2% nPVA 1% DS 5% gelatin, drying prevented by mineral oil or wet tissue. 1 Hz, 0.5% (**c**) or 1% (**a, b**) strain, representative curves of 3 separate photo-rheometry experiments (N=3) shown.

Supplementary Figure 17

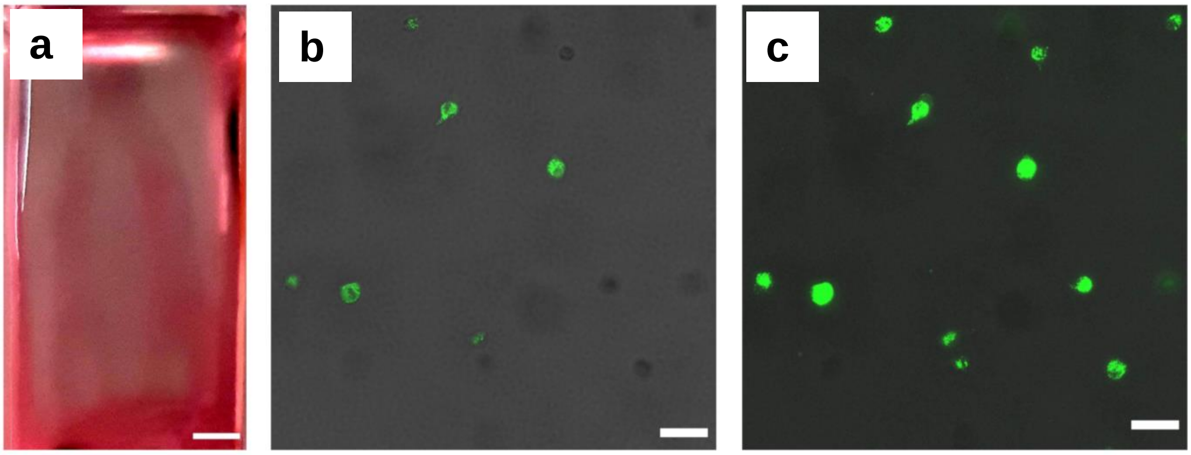

Supplementary Fig. 17: Volumetric bioprinting of PIPS resin. **a** Image of the bioprinted branch structure taken after loading medium (red, contrast enhanced for better visibility). **b-c** Representative CLSM images of hMSCs stained with Calcein AM (green) showing alive cells, transmission channel (grey) showing pores in **b**; viability seems high considering the number of cells in focus the total amount of cells; note that some cells that are seen in the images are out of focus, which can be seen well when comparing **b** (single plane) and **c** MIP of 50 µm thick z-stack. Scale bars: 2 mm (**a**) and 50 µm (**b**, **c**).

Supplementary Figure 18

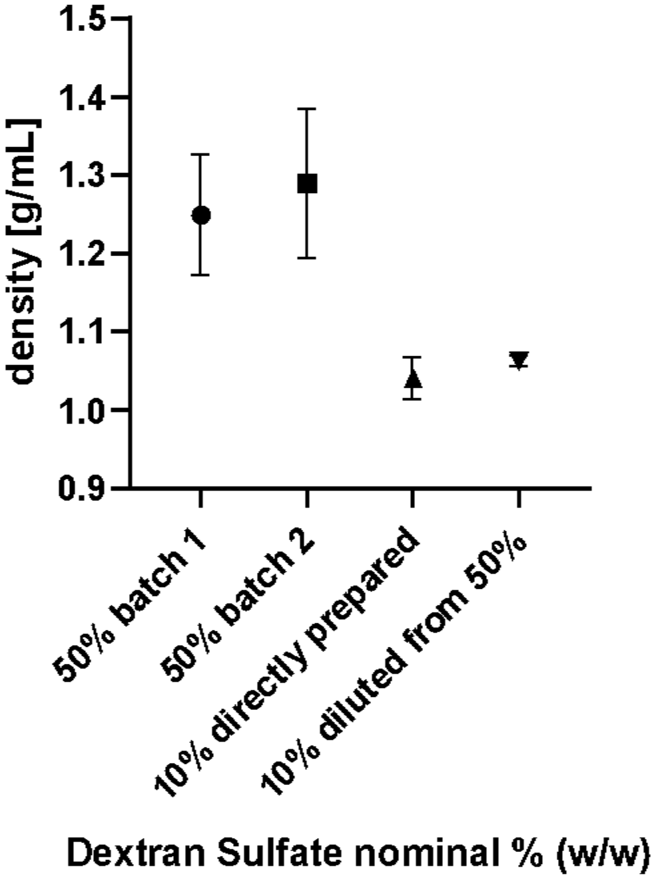

Supplementary Fig. 18: Density of different DS stock solutions. Obtained from weighing 10 µL, N=3 samples, data presented as mean ± SD.

Supplementary Figure 19

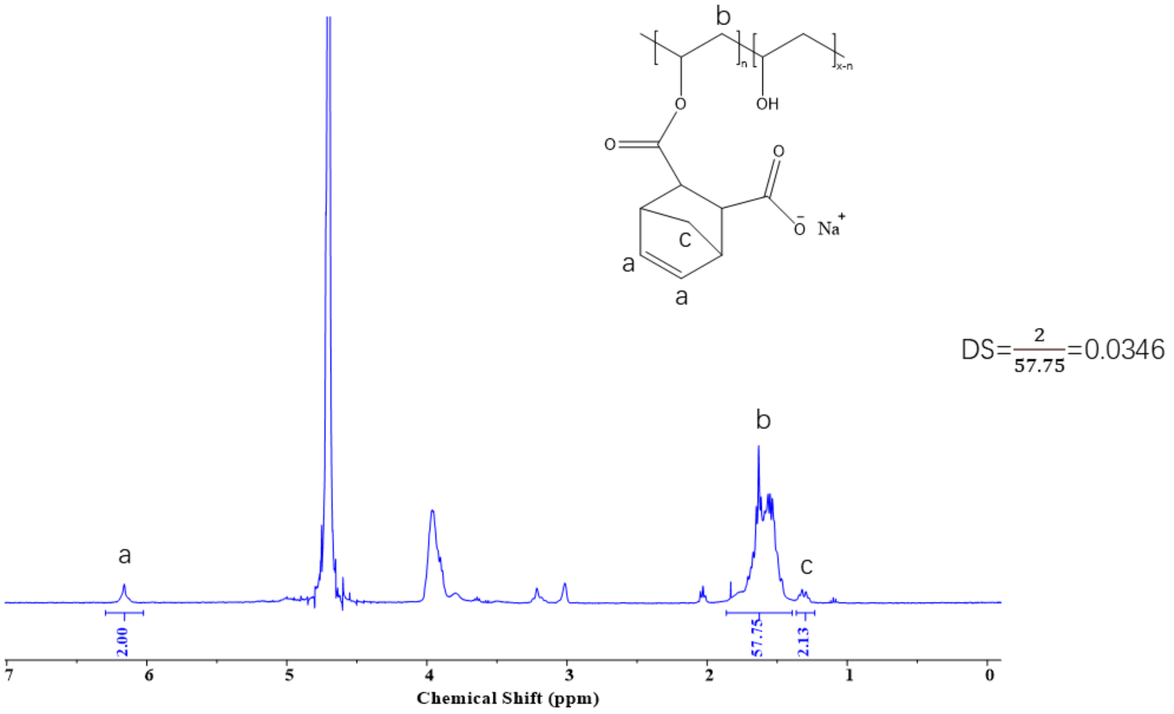

Supplementary Fig. 19: ^{1}H-NMR spectrum of nPVA in D_2O (400 MHz).

Supplementary Figure 20

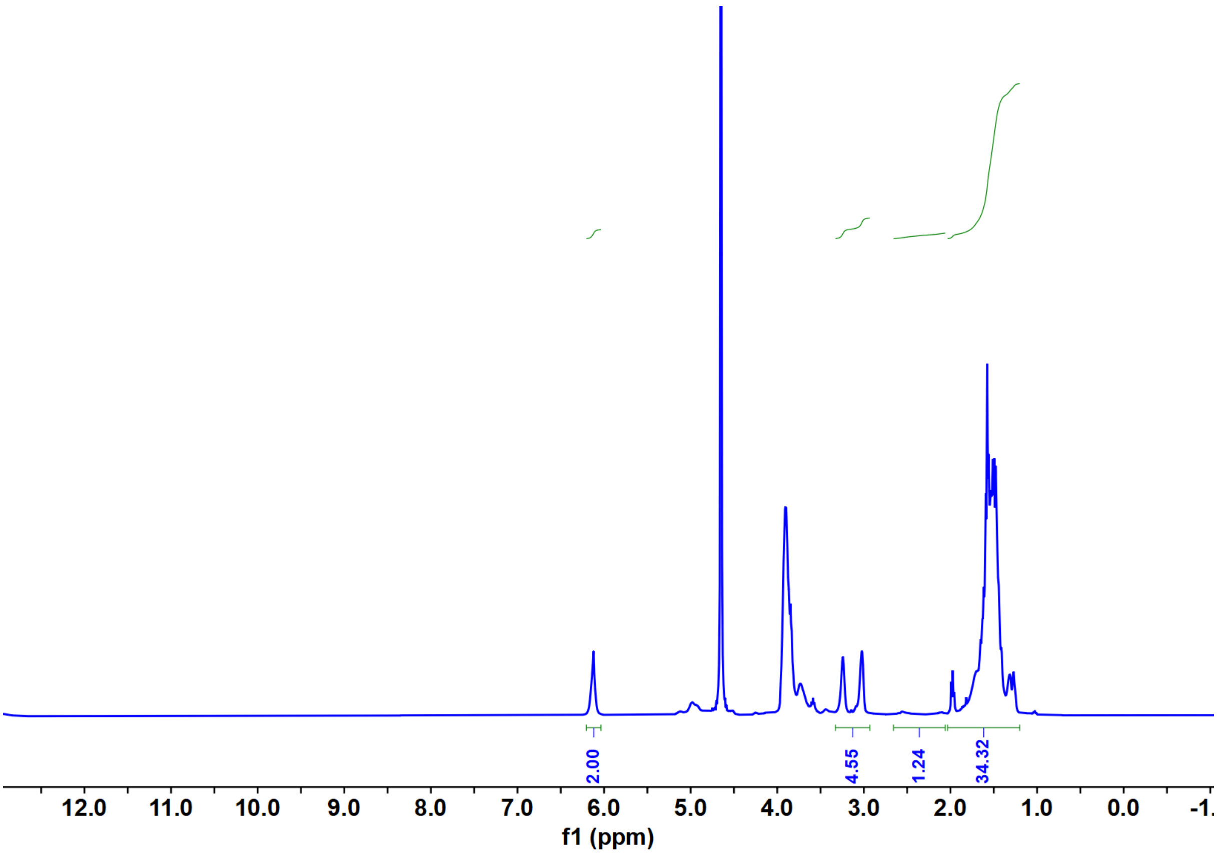

Supplementary Fig. 20: ^{1}H-NMR spectrum of nPVA-RGD conjugates in D_2O (400 MHz).

Supplementary Table 1: Results of the second laser dose test with 2% nPVA. DoF = 20% with 5% gelatin and 1% DS, green = derived threshold 75 mJ cm^{-2}.

Intensity [mW cm^{-2}]	Time [s]							
	44	39	34	29	24	19	14	9
6.6	290	257	224	191	158	125	92	59
5.8	255	226	197	168	139	110	81	52
5	220	195	170	145	120	95	70	
4.2	185	164	143	122	101	80		
3.4	150	133	116	99	82	65		
2.6	114	101	88	75	62			
1.8	79	70	61					
1	44							

Supplementary Table 2: **Components of the 2% nPVA 2.5% DS composition (Mix 1).** Other compositions with 2% nPVA but varying % DS were formulated analogously by adapting the DS and PBS relative volume fractions accordingly.

Component	Diluted in	Concentration	Relative volume [v/v]	Final concentration [w/v]
**nPVA (DoF =7%)**	0.1 % LAP	4% w/w	50%	2% (0.05% LAP)
**DS**	PBS	“10%” w/w	25%	2.5%
**PBS**		1x	12.5%	
**PEG-2-SH (2 kDa)**	PBS	15.34% w/w	12.5%	1.9% (thiol/ene ratio: 0.8)

Supplementary Table 3: Composition of the 2% nPVA 1% DS 5% gelatin resin for acellular volumetric printing (Mix 3).

Component	Diluted in	Concentration	Relative volume [v/v]	Final concentration [w/v]
**Gelatin**	PBS	10% w/v	50%	5%
**nPVA (DoF = 20%)**	0.25 % LAP	10% w/w	20%	2% (0.05% LAP)
**PBS**		1x	10%	
**DS**	PBS	10% w/w	10%	1%
**PEG-2-SH (2 kDa)**	PBS	16.09% w/w	10%	1.9%

Supplementary Table 4: Components of the 3% nPVA 3% DS composition used for cell embedding (Mix 6). Other compositions with 3% nPVA but varying % DS were formulated analogously by adapting the DS and PBS relative volume fractions accordingly.

Component	Diluted in	Concentration	Relative volume [v/v]	Final concentration [w/v]
**nPVA (DoF = 7%) + nPVA-RGD**	PBS	10% w/v	18% (total: 30%)<br>12% [a]	total: 3%
**PBS**		1x	0%[b]	
**LAP**	PBS	0.5% w/v	10%	0.05%
**DS**	PBS	10% w/v	30% [b]	3%
**PEG-2-SH (2 kDa)**	PBS, pH 6	16.51%	20%	3.3% (thiol/ene ratio: 0.8)
**Cells**	PBS	40 Mio/mL	10% [c]	4 Mio/mL

[a] for compositions without RGD, regular nPVA was used for the entire volume fraction

[b] for the control composition 3% nPVA 0% DS, the DS volume fraction was replaced with PBS

[c] for acellular experiments and controls, the cell volume fraction was replaced with PBS

MATLAB Script for FFT

```
%% FFT code

close all
clear
clc

%% Insert parameters and load image
% NB note that the image should be square!

im = imread('2021-03-05_E17.4_branch_95_pores_FITCdex_onlymerged.lif - 
25x_closetoborder.jpg');
conv = 1/1.65; %specify scalebar [Âµm/pxl] = 1/[px/um], ALWAYS CHECK IF 
TRUE 1/16.6320 (63x z3), 1/11.0880 (63xz2) 1/5.5440 (63x), 1/1.65 (25x), 
1/1.7600 (20x),1/0.8800 (10x)

%% Automatic

grayImage = rgb2gray(im); %greyscale
figure
imagesc(grayImage);
grayImage=double(grayImage);  %NEW because error using  .* otherwise
[rows columns numberOfColorChannels] = size(grayImage);
if numberOfColorChannels > 1
    grayImage = rgb2gray(grayImage);
end

% Convert from pixel size to "future" q space in 2D
matsize = size(grayImage);
L = matsize(1); %Pixel size of your image. If this is a square, matsize(1) 
and matsize(2) are the same
LL=matsize(2);
FOV = L*conv; %[Âµm] field of view of the image
Qmax = (L/2)/FOV; % Maximum wave vector
Qx = linspace(-Qmax, Qmax, L);
Qy = linspace(-Qmax, Qmax, L);

wc=hamming(L); % Make a window
[maskr,maskc]=meshgrid(wc,wc);
w=maskr.*maskc; % This is the window
fftOriginal = fft2(double(grayImage.*w)); % Take the 2d FFT of the image 
multiplied by the window

shiftedFFT = fftshift(fftOriginal); % Shift so that the centre is at the 
centre of the image
[QQx,QQy] = meshgrid(Qx,Qy);
Qr=sqrt(QQx.^2+QQy.^2);

I.fft = log10(abs(shiftedFFT));

figure
imagesc(log10(abs(shiftedFFT)),'Xdata', Qx, 'Ydata',Qy);
axis('image')
xlabel('Qx [1/um]'); ylabel('Qy [1/um]');
set(gca,'FontSize',12,'FontName','Calibri Light');
set(gcf,'color','w')
title('FFT')

% Do the radial binning
k=0;
for i=1:size(Qr,2)
    for j=1:size(Qr,1)
```

```
        k=k+1;
        PR(1,k)=Qr(i,j);
        PR(2,k)=log10(abs(shiftedFFT(i,j)));
    end
end
[~,idx] = sort(PR(:,1)');
sortedPR = PR(idx,:)';
[RBin,PropertyBin]=radial_binning(PR(1,:)',PR(2,:)',200,max(PR(1,:)));

I.RBin = RBin;
I.PropertyBin = PropertyBin;

figure
plot(RBin,PropertyBin, 'LineWidth',1.5)
xlabel('q [um^-^1]');
ylabel('I [a.u.]');
title('Radial intensity FFT')
set(gca,'FontSize',24,'FontName','Arial');
set(gcf,'color','w')
set(gca,'Yscale','log','XScale','log')
set(gca,'box','off')
set(gca,'LineWidth',1.5)
```

```
function [RBin,PropertyBin]=radial_binning(R,I,BinNo,MaxR)

%% ---------------------------------------------------------------------------
% Parameters
% ---------------------------------------------------------------------------
% Parameters of Geometry:

% Parameters for Binning:
number_of_rings = BinNo;        % number of Bins for Binning

%% ---------------------------------------------------------------------------
% Not-to-be-messed-with Parameters
% ---------------------------------------------------------------------------
R_min=0;             % Minimal Distance from Center at which
% positions are considered
R_max = MaxR; % Maximal Distance from Center at which
% positions are considered

rmin = R_min;
rmax = R_max;

Bin_Edges = linspace(rmin,rmax,number_of_rings+1);  % Edges of Bins
Bin_Centers=(Bin_Edges(1:end-1)+Bin_Edges(2:end))./2; % Center of Bins.

RadialPos=R;
Property=I;

%% ---------------------------------------------------------------------------
% Binning
%----------------------------------------------------------------------------
% The Particles are Binned according to the predefined Bins radially.

% Pre-initialize Matrices.
PropertyMean=[];

[n,whichbin] = histc(RadialPos,Bin_Edges);
for i=1:number_of_rings
    flagBinMembers=(whichbin==i);
    binMembers=Property(flagBinMembers);
```

```
    PropertyMean(i)=nanmean(binMembers);
end

%% ---------------------------------------------------------------------------
% Rename for clarity and subtract Brownian Motion
%----------------------------------------------------------------------------
PropertyBin=PropertyMean;
RBin=Bin_Centers;

end
```

References

1 Qin, X.-H., Labuda, K., Chen, J., Hruschka, V., Khadem, A., Liska, R., Redl, H. & Slezak, P. Development of Synthetic Platelet-Activating Hydrogel Matrices to Induce Local Hemostasis. *Advanced Functional Materials* **25**, 6606-6617, (2015).

2 Vandaele, J., Louis, B., Liu, K., Camacho, R., Kouwer, P. H. J. & Rocha, S. Structural characterization of fibrous synthetic hydrogels using fluorescence microscopy. *Soft Matter* **16**, 4210-4219, (2020).

3 Bernero, M., Zauchner, D., Müller, R. & Qin, X.-H. Interpenetrating network hydrogels for studying the role of matrix viscoelasticity in 3D osteocyte morphogenesis. *Biomaterials Science* **12**, 919-932, (2024).
