# Supplementary material for: Cell-guiding microporous hydrogels by photopolymerization-induced phase separation
Source: Nat Commun. 2025 May 27;16:4923. doi: 10.1038/s41467-025-60113-9 (PMC12116776; doi:10.1038/s41467-025-60113-9)
Supplement: Supplementary file 2 — Description of Additional Supplementary Files [file 41467_2025_60113_MOESM2_ESM.pdf]

Description of Additional Supplementary Files

File name: Supplementary Movie 1
Description: Animation of PIPS hydrogels with FITC-dextran-labelled porous microstructure. Scale bar: 20 µm.

File name: Supplementary Movie 2
Description: Animation of actin-nuclei-stained hMSC cells in PIPS hydrogels at day 14 following up in vitro osteogenic culture. Scale bar: 50 µm.

File name: Supplementary Movie 3
Description: Moving through a z-stack of actin-nuclei-stained hMSC cells in a FITC-dextran perfused PIPS hydrogel at day 1 of osteogenic culture. Scale bar: 50 µm.

File name: Supplementary Movie 4
Description: Volumetric printing process of the resin supplemented with gelatin within 12 seconds.
