# Supplementary material for: Cell-guiding microporous hydrogels by photopolymerization-induced phase separation
Source: Nat Commun. 2025 May 27;16:4923. doi: 10.1038/s41467-025-60113-9 (PMC12116776; doi:10.1038/s41467-025-60113-9)
Supplement: Supplementary file 7 — Reporting Summary [file 41467_2025_60113_MOESM7_ESM.pdf]

nature portfolio

Corresponding author(s): Xiao-Hua Qin

Last updated by author(s): May 13, 2025

Reporting Summary

Nature Portfolio wishes to improve the reproducibility of the work that we publish. This form provides structure for consistency and transparency in reporting. For further information on Nature Portfolio policies, see our Editorial Policies and the Editorial Policy Checklist.

Statistics

For all statistical analyses, confirm that the following items are present in the figure legend, table legend, main text, or Methods section.

n/a	Confirmed	
☐	☒	The exact sample size (*n*) for each experimental group/condition, given as a discrete number and unit of measurement
☐	☒	A statement on whether measurements were taken from distinct samples or whether the same sample was measured repeatedly
☐	☒	The statistical test(s) used AND whether they are one- or two-sided<br>*Only common tests should be described solely by name; describe more complex techniques in the Methods section.*
☒	☐	A description of all covariates tested
☐	☒	A description of any assumptions or corrections, such as tests of normality and adjustment for multiple comparisons
☐	☒	A full description of the statistical parameters including central tendency (e.g. means) or other basic estimates (e.g. regression coefficient) AND variation (e.g. standard deviation) or associated estimates of uncertainty (e.g. confidence intervals)
☐	☒	For null hypothesis testing, the test statistic (e.g. *F*, *t*, *r*) with confidence intervals, effect sizes, degrees of freedom and *P* value noted<br>*Give P values as exact values whenever suitable.*
☒	☐	For Bayesian analysis, information on the choice of priors and Markov chain Monte Carlo settings
☒	☐	For hierarchical and complex designs, identification of the appropriate level for tests and full reporting of outcomes
☒	☐	Estimates of effect sizes (e.g. Cohen's *d*, Pearson's *r*), indicating how they were calculated

Our web collection on statistics for biologists contains articles on many of the points above.

Software and code

Policy information about availability of computer code

Data collection	Leica LAS X v2.0.0.14332.2, ImageJ v1.54d, MATLAB R2018a, Imaris v9.9, Rstudio/R 4.1.1, https://github.com/BorisLouis/Segmentation/
Data analysis	GraphPad Prism v.9.2.0, Microsoft Excel v16.0

For manuscripts utilizing custom algorithms or software that are central to the research but not yet described in published literature, software must be made available to editors and reviewers. We strongly encourage code deposition in a community repository (e.g. GitHub). See the Nature Portfolio guidelines for submitting code & software for further information.

Data

Policy information about availability of data

All manuscripts must include a data availability statement. This statement should provide the following information, where applicable:

- Accession codes, unique identifiers, or web links for publicly available datasets
- A description of any restrictions on data availability
- For clinical datasets or third party data, please ensure that the statement adheres to our policy

The data that support the findings of this study are available in the ETH Zurich Research Collection with the identifier [DOI: 10.3929/ethz-b-000734397]. Data is available from the corresponding author on request.

Research involving human participants, their data, or biological material

Policy information about studies with human participants or human data. See also policy information about sex, gender (identity/presentation), and sexual orientation and race, ethnicity and racism.

Reporting on sex and gender	n/a
Reporting on race, ethnicity, or other socially relevant groupings	n/a
Population characteristics	n/a
Recruitment	n/a
Ethics oversight	n/a

Note that full information on the approval of the study protocol must also be provided in the manuscript.

Field-specific reporting

Please select the one below that is the best fit for your research. If you are not sure, read the appropriate sections before making your selection.

[x] Life sciences [ ] Behavioural & social sciences [ ] Ecological, evolutionary & environmental sciences

For a reference copy of the document with all sections, see nature.com/documents/nr-reporting-summary-flat.pdf

Life sciences study design

All studies must disclose on these points even when the disclosure is negative.

Sample size	No statistical methods were used to determine sample size. Experiments were conducted with a minimum sample size of n=3 independent replicates. For image quantifications, multiple fields of view were used per replicate. Sample size was selected following common practice in the field (e.g. Broguiere et al., Biomaterials 200:56-65 (2019)).
Data exclusions	No data were excluded.
Replication	The outcomes obtained from acellular experiments and 3D hydrogel culture were consistent and replicable in at least two independent experiments, with a minimum of three replicates in each experiment. An exception was the proof-of-concept osteogenic culture of 28 days, performed once.
Randomization	This study was not randomized. The experiments did not include assigning samples to experimental and control groups, instead, different compositions were compared.
Blinding	This study was not blinded. Blinding was not feasible due to the often visible difference between compositions. However, all quantitative data were acquired using automated image analysis pipelines and standardized methods, minimizing the risk of observer bias.

Reporting for specific materials, systems and methods

We require information from authors about some types of materials, experimental systems and methods used in many studies. Here, indicate whether each material, system or method listed is relevant to your study. If you are not sure if a list item applies to your research, read the appropriate section before selecting a response.

Materials & experimental systems

n/a	Involved in the study	
[ ]	[x]	Antibodies
[ ]	[x]	Eukaryotic cell lines
[x]	[ ]	Palaeontology and archaeology
[x]	[ ]	Animals and other organisms
[x]	[ ]	Clinical data
[x]	[ ]	Dual use research of concern
[x]	[ ]	Plants

Methods

n/a	Involved in the study	
[x]	[ ]	ChIP-seq
[x]	[ ]	Flow cytometry
[x]	[ ]	MRI-based neuroimaging

Antibodies

Antibodies used	Anti-Osteocalcin (dilution 1:200, Abcam, ab93876, rabbit polyclonal)<br>Anti-Collagen I (dilution 1:200, Abcam, ab6308, mouse monoclonal)<br>Alexa Fluor 488 (dilution 1:500, Invitrogen, A21202, donkey anti-mouse IgG H&L polyclonal)<br>Alexa Fluor 555 (dilution 1:500, Abcam, ab150082, goat anti-rabbit IgG H&L, polyclonal)
Validation	All antibodies utilized underwent validation across various research reports, with specific references provided for each primary antibody. Only antibodies that were certified and validated by reputable companies were employed for immunohistochemistry (IHC). The cellular localization of all proteins investigated had been previously documented and aligned with the expressions reported in this study. Antibody staining was additionally validated by a secondary antibody control without adding the primary antibody.

Eukaryotic cell lines

Policy information about cell lines and Sex and Gender in Research

Cell line source(s)	Human bone marrow-derived mesenchymal stem cells (hMSCs), Lonza PT-2501 (Lot# 19TL281098, 25 year-old male)<br>Human dermal fibroblasts (HDFs), Cell Applications Inc. 106-05a (Lot# 1526, 57-year-old female)
Authentication	All commercial cell lines have been authenticated by the supplier.
Mycoplasma contamination	All cell lines tested negative for mycoplasma contamination.
Commonly misidentified lines<br>(See ICLAC register)	No commonly misidentified cell lines listed by the ICLAC register were used in this work.

Plants

Seed stocks	n/a
Novel plant genotypes	n/a
Authentication	n/a
